# Sudden Cardiac Death in Anabolic-Androgenic Steroid Users: A Literature Review

**DOI:** 10.3390/medicina56110587

**Published:** 2020-11-04

**Authors:** Marco Torrisi, Giuliana Pennisi, Ilenia Russo, Francesco Amico, Massimiliano Esposito, Aldo Liberto, Giuseppe Cocimano, Monica Salerno, Giuseppe Li Rosi, Nunzio Di Nunno, Angelo Montana

**Affiliations:** 1 Legal Medicine, Department of Medical, Surgical and Advanced Technologies, “G.F. Ingrassia”, University of Catania, 95123 Catania, Italy; marcotorrisi1992@gmail.com (M.T.); giu.pennisi@gmail.com (G.P.); ileniarusso@hotmail.it (I.R.); francescoamico08@gmail.com (F.A.); massimiliano.esposito91@gmail.com (M.E.); aldoliberto@gmail.com (A.L.); peppecocimano@hotmail.it (G.C.); monica.salerno@unict.it (M.S.); 2 Department of Law, Criminology, Magna Graecia University of Catanzaro, 88100 Catanzaro, Italy; lirosigiose@gmail.com; 3 Department of History, Society and Studies on Humanity, University of Salento, 73100 Lecce, Italy; nunzio.dinunno@icloud.com

**Keywords:** AASs, anabolic androgenic steroids, SCD, sudden cardiac death, cardiac damage, adverse effects, cardiac toxicity

## Abstract

*Background and objectives:* Anabolic-androgenic steroids (AASs) are a group of synthetic molecules derived from testosterone and its related precursors. AASs are widely used illicitly by adolescents and athletes, especially by bodybuilders, both for aesthetic uses and as performance enhancers to increase muscle growth and lean body mass. When used illicitly they can damage health and cause disorders affecting several functions. Sudden cardiac death (SCD) is the most common medical cause of death in athletes. SCD in athletes has also been associated with the use of performance-enhancing drugs. This review aimed to focus on deaths related to AAS abuse to investigate the cardiac pathophysiological mechanism that underlies this type of death, which still needs to be fully investigated. *Materials and Methods:* This review was conducted using PubMed Central and Google Scholar databases, until 21 July 2020, using the following key terms: “((Sudden cardiac death) OR (Sudden death)) AND ((androgenic anabolic steroid) OR (androgenic anabolic steroids) OR (anabolic-androgenic steroids) OR (anabolic-androgenic steroid))”. Thirteen articles met the inclusion and exclusion criteria, for a total of 33 reported cases. *Results:* Of the 33 cases, 31 (93.9%) were males while only 2 (61%) were females. Mean age was 29.79 and, among sportsmen, the most represented sports activity was bodybuilding. In all cases there was a history of AAS abuse or a physical phenotype suggesting AAS use; the total usage period was unspecified in most cases. In 24 cases the results of the toxicological analysis were reported. The most detected AASs were nandrolone, testosterone, and stanozolol. The most frequently reported macroscopic alterations were cardiomegaly and left ventricular hypertrophy, while the histological alterations were foci of fibrosis and necrosis of the myocardial tissue. *Conclusions:* Four principal mechanisms responsible for SCD have been proposed in AAS abusers: the atherogenic model, the thrombosis model, the model of vasospasm induced by the release of nitric oxide, and the direct myocardial injury model. Hypertrophy, fibrosis, and necrosis represent a substrate for arrhythmias, especially when combined with exercise. Indeed, AAS use has been shown to change physiological cardiac remodeling of athletes to pathophysiological cardiac hypertrophy with an increased risk of life-threatening arrhythmias.

## 1. Introduction

AASs are a group of synthetic molecules derived from testosterone and its related precursors. AAS were developed to minimize the androgenic effects of testosterone and maximize the anabolic effects promoting the growth of skeletal muscles [1,2,3]. AASs can be administered orally, by intramuscular or subcutaneous injection, by pellet subcutaneous implantation, or by application on the skin.

Only a few AAS are used or proposed for therapeutic use, mainly in replacement treatment of hypogonadism [4,5]. Direct testosterone replacement therapy (TRT) is the only FDA-approved therapy for the treatment of male hypogonadism [6]. Oxandrolone, instead, is used to fight the protein catabolism associated with long-term use of corticosteroids and in the treatment of bone pain due to osteoporosis [7]. Clinical studies have demonstrated the efficacy of oxandrolone in the treatment of acute catabolic disorders, such as severe burns or severe trauma, and chronic catabolic disorders such as AIDS-associated cachexia or neuromuscular diseases such as Duchenne muscular dystrophy [8]. As well as for therapeutic use, AAS are widely used illicitly by adolescents and athletes, especially by bodybuilders, both for aesthetic uses and as performance enhancers to increase muscle growth and lean body mass, in consideration of their significant anabolic effect [9,10].

Although the use of AASs is now widespread around the world, with around ten million users, there are some geographical differences. The Middle East has the highest prevalence rate, with 21.7% of world users, followed by South America (4.8%), Europe (3.8%), North America (3.0%), Oceania (2.6%), Africa (2.4%), and Asia (0.2%). Among developed countries, the highest prevalence is found in Scandinavia, the United States, and countries of the British Commonwealth. The highest overall prevalence rate of AAS use was found in recreational sportspeople (18.4%), followed by athletes (13.4%), prisoners (12.4%), and drug users (8.0%). Non-athletes have the lowest prevalence rate that is estimated to be about 1% [11,12]. The global lifetime prevalence rate of AAS use is estimated to be 3.3%, greater in men than women (6.4% vs. 1.6%). As concerns the age of AAS users, teenagers have a higher overall prevalence rate (2.5%) than people older than 19 years (1.9%). The prevalence rate among high-school students was 2.3% [11,13,14].

While the use of AASs for medical indications is relatively safe, when used illicitly they can damage health and cause disorders affecting several functions (cardiovascular, reproductive, musculoskeletal, endocrine, renal, immunologic, and neuropsychiatric) [3,15,16,17,18,19,20,21,22,23]. These side effects include cardiac injuries such as fibrosis, cardiac hypertrophy, and dilated cardiomyopathy with an increased risk for myocardial infarction, arrhythmias, and sudden cardiac death [3,24,25,26,27].

AAS are typically used in phases referred to as “cycles”. To reduce the dangerous consequences of continuous AAS use at supraphysiological doses, abusers often introduce changes to their intake [28,29]. “Stacking” consumption can also involve nutritional supplements, complements, or other substances [30,31,32]. The substances most frequently taken at the same time as AASs are alcohol, amphetamines, aspirin, cannabinoids, caffeine, clomiphene citrate, cocaine, codeine, creatine, ephedrine, erythropoietin, furosemide, gamma-hydroxybutyrate (GHB), growth hormone, heroin, insulin, insulin-like growth 1 (IGF-1), melanotan, protein powder, tamoxifen, thyroxine, and tobacco. [33].

Sudden cardiac death (SCD) is generally defined as a sudden unexpected death or arrest from a presumed cardiac cause, which occurs within one hour of symptom onset if witnessed, otherwise within 24 h, in a person without any prior condition that would appear fatal [34,35,36].

This review aims to investigate the relationship between the use of anabolic-androgenic steroids (AAS) and sudden cardiac death in athletes and identify the possible etiological mechanism.

## 2. Materials and Methods

### 2.1. Database Search Terms and Timeline

This review was conducted performing a systematic literature search on online resources (PubMed Central database and Google Scholar) until 21 July 2020, using the following key terms: “((Sudden cardiac death) OR (Sudden death)) AND ((androgenic anabolic steroid) OR (androgenic anabolic steroids) OR (anabolic-androgenic steroids) OR (anabolic-androgenic steroid))”.

### 2.2. Inclusion and Exclusion Criteria

The following inclusion criterion were applied: full-text scientific article published in English. The following exclusion criteria were adopted: (1) conference abstracts or reviews and letters to the editor without case reports; (2) animal studies; (3) articles in which the correlation between cardiac death and steroids is not discussed; (4) articles regarding surviving subjects.

### 2.3. Study Selection

**Figure 1:**
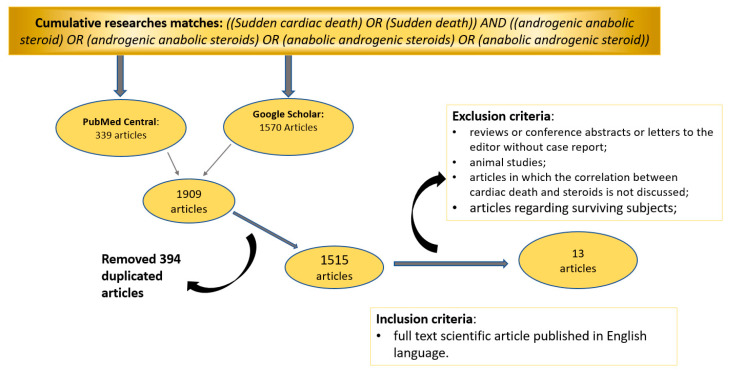
Flow diagram with inclusion and exclusion criteria for the selection of sources for the purpose of the review.

We retrieved 1909 articles (339 from Pubmed Central and 1570 from Google Scholar databases). After excluding all duplicate articles, the reviewers retrieved abstracts and full text of each article independently applying the inclusion and exclusion criteria. Figure 1 summarizes the data obtained after our literature search.

## 3. Results

The review of the literature using the flow diagram shown in Figure 1 allowed us to identify 13 articles (Table 1) published between 1993 and 2020, for a total of 33 reported cases. The main characteristics of each selected article are summarized in Appendix A.

Of the 33 cases, 31 (93.9%) were males while only 2 (6.1%) were females. The mean age was 29.79 years with an SD of 8.5 years (range 13–54). Twenty-one cases (63.6%) were sportsmen and the most represented sports activity was bodybuilding (13 cases, 39%).

In all cases, there was a history of AAS abuse or a physical phenotype suggesting AAS use, the total period of AAS use was unspecified in 24 cases. In the other 9 cases, the time in which the subjects took AAS varied from 3 months to several years. In 24 cases the results of the toxicological analysis were reported. The tests were negative in 4 subjects. In 8 individuals, the toxicological examination revealed the presence of one or more AASs (Table 2) in blood or urine. The most detected AASs were nandrolone (10 cases), testosterone (9 cases), and stanozolol (7 cases). In two cases the toxicological examination found the presence of other substances used as performance-enhancing drugs (clenbuterol, ephedrine, norephedrine, liothyronine). In five other cases, other drugs of abuse were found such as cocaine, opioids, benzodiazepines, and cannabinoids.

In 15 cases it was possible to calculate the weight of the heart as a percentage of body weight (Appendix A). Anamnestic data were present in 24 of the 33 cases examined (72.7%). Of these, in no case was there a personal history of the disease or a family history of heart disease before age 50.

**Figure 2:**
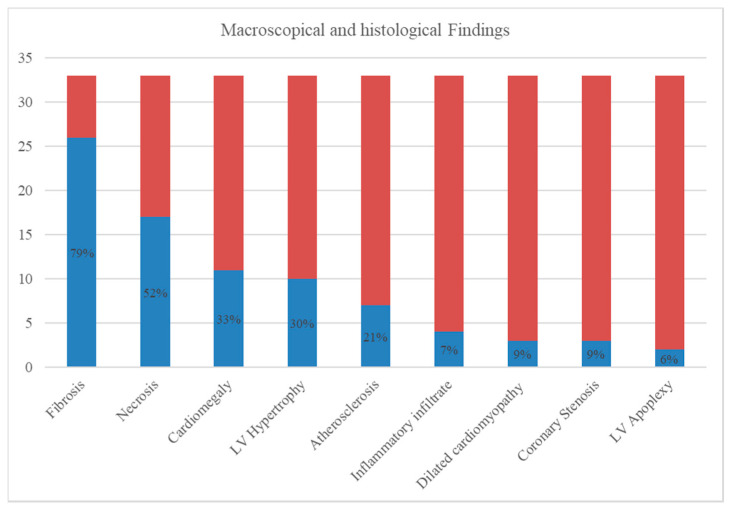
Summary of macroscopic and histologic findings.Autopsies ruled out the causes of extracardiac death in all cases. However, in most cases (20.61%) it was not possible to define the exact cardiac cause that led to death, although SCD was correlated with the use of AASs in all cases.

In the 33 examined cases, the most frequent macroscopic alteration was cardiomegaly (11 cases, 33%), based on the weight of the heart as a percentage of body weight, followed by left ventricular hypertrophy (10 cases, 30%). Dilated cardiomyopathy was found in 3 cases (9%). The most frequently reported histological alteration were foci of fibrosis and necrosis of the myocardial tissue, found respectively in 21 (79%) and 17 cases (52%). Other histological alterations reported were atherosclerosis (7 cases, 21%), inflammatory infiltrate (4 cases, 12%), coronary stenosis (3 cases, 9%), and left ventricular apoplexy (2 cases, 6%). The macroscopic and histological findings are summarized in Figure 2.

## 4. Discussion

Data emerging from our study confirm the higher prevalence of ASS assumption among young males (93.9% males compared to 6.1%females, mean age 29.79 years), especially if they are bodybuilders (39%). In none of the cases in which anamnestic data were present was there a personal history of the disease or a family history of heart disease before age 50. In the 33 cases examined, the most frequently reported macroscopic changes were cardiomegaly (33%) and left ventricular hypertrophy (30%). The most frequently reported histological changes were foci of fibrosis (79%) and necrosis (52%) of myocardial tissue. In all cases, autopsies ruled out causes of extracardiac death, and SCD was correlated with AAS use. Sudden cardiac death (SCD) is generally defined as a sudden unexpected death or arrest from a presumed cardiac cause, which occurs within one hour of symptom onset if witnessed, otherwise within 24 h, in a person without any prior condition that would appear fatal [34,35,36]. SCD in athletes is an event that profoundly impacts society because athletes are generally seen as a healthy category of people. Although SCD is the most common medical cause of death in athletes, its true incidence is unknown. The risk of SCD in athletes is 2 to 3 times greater than that in the general population. This difference may be due to typical athletes' demographic factors, such as sex, age, and ethnicity. Potential mechanisms for SCD consist of inflammation, mechanical factors such as ventricular hypertrophy or fibrosis, neurological and metabolic comorbidities, and hereditary factors, arrhythmic mechanisms of abnormal ventricular repolarization, conduction, or autonomic innervation [49]. The etiology of SCD in younger athletes (<35 years of age) is mainly related to inherited cardiac conditions, instead, in older athletes, it is related to atherosclerotic coronary artery disease (CAD) [50,51,52,53]. Left ventricular hypertrophy (LVH) has been recognized as an independent risk factor for sudden cardiac death. The high mortality and sudden cardiac death associated with LVH is related to ventricular arrhythmia. Indeed, hypertrophied myocardium has a typical pro-arrhythmic electrophysiological phenotype and predisposes to the presence of myocardial ischemia. The main abnormality is prolongation of the action potential duration and refractoriness, which represents the substrate for arrhythmias [54]. Increased risk of ventricular arrhythmias and SCD, associated with hypertrophy, are related to complex processes involving myocardial cells, interstitium, coronary flow reserve, and neurohumoral activation [55]. SCD in athletes has also been associated with the use of performance-enhancing drugs, both anabolic-androgenic steroids and nonsteroidal agents [56]. AAS users often combine the assumption of anabolic substances with other substances such as cocaine, methamphetamine, and smart drugs. These data are in agreement with the results of our review. Mixing two or more substances increases the risk of negative drug interactions, worsening any adverse effects, including SCD [17].

The higher prevalence of AAS use among athletes, especially non-professionals, can be explained by their determination to achieve a perfect body and to improve performance and self-esteem. Indeed, the positive effects of AAS use are the increase of muscle mass, strength, energy and concentration, and the reduction of fat mass [57,58]. However, the use of anabolic-androgenic steroids has also many negative effects. Many of these are mild and transient (fluid retention, acne, agitation, gynecomastia, aggressiveness), but others are more serious and can damage multiple organs and functions, such as cardiovascular, reproductive, musculoskeletal, endocrine, renal, immunologic, and neuropsychiatric functions [2,57,58,59,60]. The cardiovascular system is one of the most affected by the side effects of AAS use. AAS use enhances vascular resistance and blood pressure, pro-inflammatory biomarker profile, and sympathetic tone alters serum lipoproteins and produces direct myocardial toxicity [53,61]. The adverse cardiovascular events reported are: impaired left ventricular function, arterial thrombosis, pulmonary embolism, and left ventricular hypertrophy, associated with myocytolysis and fibrosis [1]. It is reported that AAS abuse can promote cardiac tissue growth, leading to hypertrophic cardiomyopathy, followed by apoptotic cell death. This phenomenon is associated with ventricular remodeling, cardiomyopathy, myocardial infarction, and SCD and can explain how AAS may lead to cardiac death without coronary thrombosis or atherosclerosis [62,63]. AASs cause cardiac hypertrophy by a direct action on cardiac androgen receptors and these effects are directly proportional to the dose, time, and duration of administration [45]. Melchert and Welder proposed at least four hypothetical models explaining how AASs cause cardiovascular side effects. The atherogenic model concerns the alterations on lipoprotein serum levels caused by AASs, increasing the risk of atherosclerosis. The thrombosis model regards enhancing platelet aggregation and polycythemia that increase the risk of thrombus formation. The third model involves vasospasm caused by nitric oxide release induced by anabolic agents. The direct myocardial injury model concerns direct myocardial toxicity causing apoptosis, with increased collagen deposition, fibrosis, and altered microcirculation resulting in chronic ischemic damage. All of these mechanisms associate AAS use with a high risk of SCD [64,65].

A recent study showed that chronic nandrolone treatment with or without severe training causes a significant increase in beta–myosin heavy chain (β-MHC) gene expression, calcium/calmodulin-dependent protein kinaseIIδ (CaMKIIδ), and monoamine oxidase (MAO) activities in the heart tissue of male Wistar rats [66]. Cardiac hypertrophy has a genetic substrate too; ND, in adult rats, reduces cardiac contractile performance through enhancing β-MHC mRNA expressions, causing alterations of pressure-overload cardiac hypertrophy [67].

In the 9 cases of SCD, the most representative macroscopic alterations were cardiomegaly and left ventricular hypertrophy. Cardiomegaly was diagnosed by comparing the weight of the heart with the body weight and BMI of the subject [68,69,70]. Histologically, the most representative alterations were fibrosis and necrosis. These results are in agreement with what has been reported by many authors, according to whom myocardial necrosis and focal myocardial fibrosis, are highly significant alterations in the hearts of athletes who abuse AAS and may be responsible for atrioventricular conduction abnormalities and provide a substrate for the occurrence of potentially lethal arrhythmias and SCD [40,71,72,73]. It must be taken into account that, regardless of AAS abuse, increased cavity dimensions, wall thickness, and left ventricular mass are typical consequences of high-intensity exercise training and are included in the physiological cardiac remodeling of the “athlete’s heart”. A modest amount of fibrosis may be present in physiological cardiac remodeling associated with lifelong endurance training. This fibrosis and hypertrophy represent a substrate for arrhythmias [74,75]. When combined with exercise, AAS use has been shown to change physiological cardiac remodeling of the athlete to pathophysiological cardiac hypertrophy with an increased risk of life-threatening arrhythmias [1,76].

It is difficult to distinguish the etiology of these changes from histological findings alone, and it becomes essential to evaluate the subject’s clinical history and physical characteristics in all cases of sudden cardiac death in which AAS abuse is suspected. The physical phenotype of a male who abuses AASs includes some characteristics such as muscle hypertrophy, prominence striae above the pectoralis or biceps muscle, breast development in men (gynecomastia), testicular atrophy, and severe acne that may suggest AAS abuse [1,77]. In women, signs of AAS use also include hirsutism, deepening of the voice, and masculinization of secondary sexual characteristics [78,79].

Long-term use of AASs causes cardiac alterations affecting the conductive system, as demonstrated by subjects who have undergone signal-averaging electrocardiography (SAECG). SAECG is an inexpensive, safe, and highly reproducible technique that records low-amplitude electrical activity in the myocardium and provides information on the presence of a monomorphic TV substrate. SAECG performed on AAS users shows alterations in myocardial electrophysiology such as significantly longer QTc interval and greater QT dispersion, at rest and after moderate exercise, and attenuated heart rate recovery after exercise compared to subjects who do not use AASs. Abnormal SAECG indicates a re-entry mechanism for arrhythmias and, because sympathetic thrust during acute exercise lowers the ventricular fibrillation threshold, these individuals will be at increased risk of tachyarrhythmia and potential SCD following exercise [73,80,81]. Despite evidence linking the use of AAS abuse to SCD, reports in the literature are most likely underestimated due to the few autopsy data and because of the study of the pathophysiological mechanisms that lead to sudden cardiac death in subjects using AASs is severely limited [44,48,53]. In fact, information on the modalities and doses relating to the abuse of AASs is generally self-reported. Furthermore, most of the data in the literature on the effects of AAS administration derive from animal studies, as the administration of high doses of AASs in humans would be unethical, given the serious health risks [82].

Because of the high prevalence of AAS use among athletes, toxicological investigations are therefore fundamental in those cases of sudden death in subjects suspected of consuming AASs [83,84]. To date, there are still few studies published in the literature that correlates SCD in athletes with AAS use, highlighting the pathophysiological mechanisms that cause it and that are mostly based on experimental models derived from animal experiments [73,76,82,85,86,87,88,89,90,91]. It could be important to investigate new research fields to define the exact mechanism of action. For this reason, in recent years some studies have been carried out on miRNAs, a family of non-coding nucleotides that control gene expression and that appear to be related to numerous diseases. Mir-133a and mir-1, for example, appear to increase the risk of arrhythmia in the ischemic heart and may, in the future, play a role as prognostic biomarkers [25,92,93]. Nowadays, there are no studies in the literature that link the expression of miRNAs with SCD in AAS abusers.

## 5. Conclusions

Because of the high prevalence of AAS use among athletes, toxicological investigations are therefore fundamental in those cases of sudden death in which there is suspicion of AAS consumption. The cardiovascular system is one of the most affected by the side effects of AAS use. AAS use enhances vascular resistance and increases blood pressure, pro-inflammatory biomarker profile, sympathetic tone, alters serum lipoproteins, and produces direct myocardial toxicity. In agreement with the evidence in the literature, the most reported macroscopic heart changes reported in our review were cardiomegaly and hypertrophy, and the main histological changes were necrosis of myocardial tissue and foci of fibrosis. Hypertrophy, fibrosis, and necrosis represent a substrate for arrhythmias, especially when combined with exercise. AAS use has been shown to change physiological cardiac remodeling of athletes to pathophysiological cardiac hypertrophy with an increased risk of life-threatening arrhythmias. The evaluation of the parameters of electrocardiographic repolarization at rest and post-exercise, using SAECG, could provide diagnostic and prognostic information on the risk of cardiac arrhythmias and SCD in apparently healthy subjects who chronically use supraphysiological doses of AAS [27,81].

Since the pathophysiological mechanisms that lead to SCD in subjects who use AAS have not yet been fully clarified, the link between AAS abuse and SCD is probably underestimated, considering the few data in the literature.

Toxicological investigations, performed on different matrices, such as blood, urine, and hair, can confirm the use of AAS or other drugs that may have played a role in the death. A complete autopsy with histological and immunohistochemical studies, with a particular regard to the organs in which anabolic adverse events occur most frequently, is mandatory to evaluate the relationship between AAS use and SCD.

Given the young age of the subjects who usually use AASs and given the importance of the consequences related to their abuse, the identification of new tools to study AAS use, such as miRNAs, could be an important goal for the scientific community.

Nowadays, clinicians must pay attention to indicative signs of AAS use, considering those physical and epidemiological characteristics that can lead to the suspicion of abuse of these drugs to implement primary prevention measures of the serious adverse effects of AAS use. An interesting challenge would be to further investigate these findings to be able to use these biomarkers both to facilitate the post-mortem diagnosis of sudden deaths related to AAS abuse and as a screening method in living subjects to prevent fatal consequences.

## Figures and Tables

**Table 1:** Articles included.

Author	Year	Number of Cases	Study Type
Campbell, S.E. et al. [37]	1993	1	Case report
Dickerman, R.D. et al. [38]	1995	1	Case report
Hausmann, R. et al. [39]	1998	1	Case report
Fineschi, V. et al. [40]	2001	2	Case series
Fineschi, V. et al. [41]	2007	1 ^1^	Case series
Di Paolo, M. et al. [42]	2007	4	Letter to the editor
Fanton, L. et al. [43]	2009	6 ^2^	Retrospective study
Thiblin, I. et al. [44]	2009	1	Case report
Montisci, M. et al. [45]	2012	3 ^3^	Case series
Lusetti, M. et al. [46]	2015	6	Retrospective study
Lichtenfeld, J. et al. [47]	2016	1	Case report
Lusetti, M. et al. [48]	2018	5	Retrospective study
Hernandez-Guerra, A.I. et al. [1]	2019	1	Case report

^1^ case excluded because already present in the previous article; ^2^ only 6 out of 12 cases died of sudden cardiac death (SCD); ^3^ 3 out of 4 cases died of SCD.

**Table 2:** Anabolic-androgenic steroids (AASs) found on toxicological analysis.

Toxicological Findings	Number of Cases	% of Total Cases
Nandrolone	10	30%
Testosterone	9	27%
Stanozolol	7	21%
Boldenon	2	6%
Norandrosterone	1	3%
Mesterolone	1	3%
Methandienone	1	3%
Epitestosterone	1	3%
Nortestosterone	1	3%

## Appendix A

**Author (Year)**	**Age (Years); Sex; Height; Weight**	**BMI**	**Personal and Family Medical History**	**Kind of Sport Activity**	**ASS Reported Use—Time of Assumption**	**Use of Other Drugs**	**Circumstance of Death**	**Macroscopic Heart Findings**	**Histological Heart Findings**	**Toxicological Analysis**	**Cause of Death**
Campbell, S. E. et al. (1993)	21; M	NR	Absence of significant diseases	Bodybuilder	Testosterone; nandrolone—several months	NR	Collapse during a weight-lifting workout at the gym	530 g—Marked left and right ventricular hypertrophy	Extensive perivascular fibrosis of intramural coronary arteries—interstitial fibrosis	NR	Unspecified SCD
Dickerman, R. D. et al. (1995)	20; M; 180 cm; 100.7 kg	31.08	No past or family history of cardiac disease	Bodybuilder	Methenolone depot; veterinarian testosterone enanthate—just complete a 3-month cycle	NR	Sudden witnessed death	515 g (0.51% of body weight)—Signs of concentric left ventricular hypertrophy	Mild atherosclerosis	NR	Unspecified SCD
Hausmann, R. et al. (1998)	23; M; 192 cm; 94 kg	25.50	NR	Bodybuilder	Testosterone cyclopentilpropionate; methenolone enanthate; mesterolone—9 months	Other performance-enhancing drugs (liothyronine hydrochloride, clenbuterol hydrochloride)	Found unconscious at home in his bed	500 g (0.53% of body weight)—Cardiac hypertrophy, right ventricle dilatation, focal induration of endocardium	Enlargement and nuclear polymorphism of the left ventricular muscle fibers. Disseminated focal necrosis and interstitial fibrosis	Urine: Mesterolone, methandienone, testosterone, nandrolone, clenbuterol	Unspecified SCD
Fineschi, V. et al. (2001)	32; M; 189 cm; 90 kg	25.20	No history of disease	Bodybuilder	Testosterone propionate; nandrolone—several months	NR	Sudden loss of consciousness during a weight lifting workout	450 g (0.50% of body weight)—Normal heart measures (14 × 14 × 4 cm)—Normal valves, endocardium, and coronary arteries—one grayish zone in the left ventricle myocardium	Infarct necrosis corresponding to the grayish zone—some foci of contraction band necrosis and fibrosis	Urine: Metabolites of nandrolone, metabolites of stanozolol	SCD most likely related to adrenergic stress
	29; M; 166 cm; 72 kg	26.13	His medical history was unremarkable	Bodybuilder	Nandrolone; stanozolol—several months	NR	Found unconscious at home in his bed	390 g (0.54% of body weight)—Normal heart measures (11 × 10 × 5 cm)—Normal valves, endocardium, and coronary arteries	Occasional isolated myocardial cells with contraction band and segmentation	Urine: Metabolites of nandrolone, metabolites of stanozolol	Unspecified SCD
Fineschi, V. et al. (2005)	30; M; 178 cm; 90 kg	28.41	NR	Bodybuilder	Nandrolone decanoate—6 months	Unspecified other drugs	Sudden collapse at home	400g (0.44% of body weight) —Scattered fatty streaks in coronary arteries	Focal myocardial fibrosis	Urine: Norandrosterone. Blood: nandrolone	Unspecified SCD
Di Paolo, M. et al. (2007)	29; M; 190 cm; 127 kg	35.2	No prior history of disease. No family history of cardiac disease under the age of 50	Bodybuilder	History of use of unspecified AAS—unspecified	NR	Sudden loss of consciousness during the first minutes of a spin bike lesson	490 g (0.39% of body weight)—Normal hearth wall thickness, normal valve, normal coronary arteries	Severe epicardial interstitial fibrosis, small vessel disease	Negative	Unspecified SCD
	27; M; 190 cm; 100 kg	25.8	No prior history of disease. No family history of cardiac disease under the age of 50	Bodybuilder	History of use of unspecified AAS—unspecified	NR	Sudden illness while he was at a night club	360 g (0.36% of body weight)—Normal hearth wall thickness, normal valve, normal coronary arteries	Mild focal epicardial interstitial fibrosis, small vessel disease	Urine: Stanozolol, testosterone	Unspecified SCD
	37; F; 161 cm; 71 kg	27.4	No prior history of disease. No family history of cardiac disease under the age of 50	Bodybuilder and weight lifter	History of use of unspecified AAS—unspecified	NR	Found dead in her car	310 g (0.44% of body weight) —Normal hearth wall thickness, normal valve, normal coronary arteries	Moderate focal epicardial interstitial fibrosis, small vessel disease	Negative	Unspecified SCD
	31; M; 175 cm; 79 kg	25.8	No prior history of disease. No family history of cardiac disease under the age of 50	Bodybuilder	History of use of unspecified AAS—unspecified	NR	Found dead in his bedroom: alive 7 h before	400 g (0.51% of body weight)—Normal hearth wall thickness, normal valve, normal coronary arteries	Moderate epicardial interstitial fibrosis, small vessel disease	Urine: Stanozolol	Unspecified SCD
Fanton, L. et al. (2009)	19; M	NR	No history of cardiac disease	Weight lifter	History of use of unspecified AAS—unspecified	NR	SD during training	360 g—Left ventricle apoplexy	Multiple focal areas of necrosis, myolysis, scarring fibrosis	NR	Unspecified SCD
	22; M	NR	No history of cardiac disease	PE teacher	History of use of unspecified AAS—unspecified	NR	SD during training	520 g—Left ventricle apoplexy	Multiple focal areas of necrosis, myolysis, scarring fibrosis	NR	Unspecified SCD
	25; M	NR	No history of cardiac disease	Bodybuilder	History of use of unspecified AAS—unspecified	NR	SD during training	460 g—Disseminated myocarditis	Multiple focal areas of necrosis, myolysis, scarring fibrosis	NR	Unspecified SCD
	28; M	NR	No history of cardiac disease	Soccer player	History of use of unspecified AAS—unspecified	NR	SD during training	380 g—Disseminated myocarditis	Multiple focal areas of necrosis, myolysis, scarring fibrosis	NR	Unspecified SCD
	54; M	NR	No history of cardiac disease	Marathon runner	History of use of unspecified AAS—unspecified	NR	SD during training	410 g—Coronary thrombosis and dilated cardiomyopathy	multiple focal areas of necrosis, myolysis, scarring fibrosis	NR	Unspecified SCD
	48; M	NR	No history of cardiac disease	Marathon runner	History of use of unspecified AAS—unspecified	NR	SD during training	430 g—Left ventricle hypertrophy	Multiple focal areas of necrosis, myolysis, scarring fibrosis	NR	Unspecified SCD
Thiblin, I. et al. (2009)	29; F; 172 cm; 76 kg	25.7	No history of disease	Fitness athlete	History of use of unspecified AAS—unspecified	Unspecified other drugs	Found naked in a prone position on the floor beside her bed, with a pillow partly under her body	331 g (0.44% of body weight)—Normal heart measures—Normal coronary arteries, with an isolated flat area of fatty thickening in the proximal part of the left anterior descending (LAD) coronary artery.	Lymphocytic infiltration around several middle-sized and small intramural vessels—minimal myocardial necrosis	Blood: ephedrine, norephedrine. Urine: testosterone, metabolites of stanozolol, boldenone	Sudden cardiac arrhythmia, possibly related to the combination of an otherwise unspecified inflammatory process in the heart and the acute influence of ASS and ephedrine
Montisci, M. et al. (2012)	32; M; 180 cm; 110 kg	33.95	NR	Bodybuilder	History of use of unspecified AAS—7 years (recently withdraw)	NR	Found dead at home in his bed	450 g (0.41% of body weight)—11 × 9.5 cm—cardiomegaly, concentric left ventricular hypertrophy, normal valve, normal coronary arteries	Hypertrophic myocytes, focal disarray, interstitial and replacement fibrosis, foci of lymphoplasma cellular infiltrates (CD3+), with edema and patchy necrosis	Negative	Concentric left ventricular hypertrophy, focal acute myocarditis.
	32; M; 178 cm; 94 kg	29.67	At last screening, nonspecific repolarization changes were found at ECG	Cycler	History of use of unspecified AAS—several years	NR	SD after a dentistry visit	580 g (0.62% of body weight)—12.5 × 11 cm—Cardiomegaly, hypertrophy, biventricular dilatation, normal valve, non-obstructive LAD stenosis	Hypertrophic myocytes, foci of necrosis, replacement fibrosis, LAD 50% stenosis, fibrofatty replacement	Negative	Inflammatory dilated cardiomyopathy with subacute-chronic stages, hemorrhagic pulmonary infarction
	25; M, 185 cm; 125 kg	36.52	An ECG performed 5y before death was normal	Bodybuilder	Circumstantial finding of unspecified use of AAS—unspecified	Unspecified other performance-enhancing drugs	SD while sleeping	390 g (0.31% of body weight)—10.5 × 9.5 cm—normal hearth wall thickness, normal valve, normal coronary arteries	Inflammatory infiltrate, myocyte necrosis	Urine: Testosterone, epitestosterone, nortestosterone	Eosinophilic myocarditis
Lusetti, M. et al. (2015)	39 (mean age); M (All 6 cases)	NR	NR	NR	History of use of unspecified AAS—unspecified	NR	Sudden unwitnessed death	Normal hearth wall thickness, normal valve, normal coronary arteries. In one case: 490 g (0.54% of body weight)	Interstitial fibrosis (6 cases); perivascular fibrosis (4 cases); perineural fibrosis within the left ventricle (2 cases); fibroadipous metaplasia (2 cases); contraction band necrosis (2 cases); Myocyte segmentation (2 cases); Intercalated disc widening (2 cases); myocyte hypertrophy (3 cases); coronary intimal and media thickening (4 cases)	Blood: Ethanol (1 case). Urine and hair: nandrolone (3 cases), Testosterone (3 cases)	Sudden cardiac arrhythmia
Lichtenfeld, J. et al. (2016)	13; M	NR	No prior history of disease. An episode of syncope with exertion 1 week before cardiac arrest. No family history of sudden death, hypertrophic cardiomyopathy, or heart rhythm abnormalities	Sprinter	Physical Phenotype suggesting AAS use	NR	Sudden cardiac arrest while performing timed wind sprints at a competitive sports camp	465 g—Cardiomegaly, marked LV Hypertrophy	Foci of myofibrillar disarray, the proliferation of fibroblasts consistent with early fibrosis, and enlarged myofibers with the heterogeneity of nuclear size including “box-car” nuclei	NR	Sudden cardiac arrest followed by brain death
Lusetti, M. et al. (2018)	32; M	NR	No “officially” medically prescribed drug treatment at the time of death.	NR	History of use of unspecified AAS—unspecified	NR	unspecified SD	390 g—Left ventricular hypertrophy	Myocardial fibrosis	Urine: Nandrolone, Testosterone. Blood: Methadone, Citalopram, Clozapine, Venlafaxine, Lorazepam, Phenobarbital, THC	Unspecified SCD
	32; M	NR	No “officially” medically prescribed drug treatment at the time of death.	NR	History of use of unspecified AAS—unspecified	NR	unspecified SD	360 g	Fatty streaks, intima, and media thickening within the coronary arteries	Urine: Boldenone, Clomiphene, Methenolone, Oxandrolone, Stanozolol. Blood: Lorazepam, THC	Unspecified SCD
	33; M	NR	No “officially” medically prescribed drug treatment at the time of death.	NR	History of use of unspecified AAS—unspecified	NR	unspecified SD	425 g—Left ventricular hypertrophy	Myocyte necrosis	Urine: Testosterone. Blood: Methadone, Cocaine	Unspecified SCD
	39; M	NR	No “officially” medically prescribed drug treatment at the time of death.	NR	History of use of unspecified AAS—unspecified	NR	unspecified SD	480 g—Left and right ventricular hypertrophy	Myocyte necrosis, Myocardial fibrosis	Urine: Nandrolone. Blood: Morphine, THC	Unspecified SCD
	29; M	NR	No “officially” medically prescribed drug treatment at the time of death.	NR	History of use of unspecified AAS—unspecified	NR	unspecified SD	340 g	NR	Urine: Nandrolone, Testosterone. Blood: morphine, THC, Ethanol	Unspecified SCD
Hernandez-Guerra, A. I. et al. (2019)	24; M; 178 cm; 85 Kg	26.8	No past or family history of cardiac disease. One episode of precordial pain some months before.	NR	stanozolol, testosterone, mesterolone, nandrolone—6 months	tamoxifen	Sudden death at home	420 g (0.49% of body weight)—Cardiomegaly, Normal ventricular thickness, >75% Stenosis of the left main trunk and the LAD, areas of scarring located at the intersection between the posterior wall and the posterior third of the septum	Acute myocardial infarction, myocytes hypertrophy, small intramyocardial vessel disease	Blood: Ethanol, Stanozolol, Nandrolone	Acute myocardial infarction
